# The MARVIN Hypothesis: Linking Unhealthy Lifestyles to Intracranial Aneurysm Rupture Risk and Clinical Prognosis

**DOI:** 10.3390/medicina60111813

**Published:** 2024-11-04

**Authors:** Vanessa M. Swiatek, Igor Fischer, Rajiv Khajuria, Amir Amini, Hannah Steinkusch, Ali Rashidi, Klaus-Peter Stein, Claudia A. Dumitru, I. Erol Sandalcioglu, Belal Neyazi

**Affiliations:** 1 Department of Neurosurgery, Otto-von-Guericke University, 39120 Magdeburg, Germany; vanessa.swiatek@med.ovgu.de (V.M.S.); amir.amini@med.ovgu.de (A.A.); hannah.steinkusch@st.ovgu.de (H.S.); ali.rashidi@med.ovgu.de (A.R.); klaus-peter.stein@med.ovgu.de (K.-P.S.); claudia.dumitru@med.ovgu.de (C.A.D.); erol.sandalcioglu@med.ovgu.de (I.E.S.); 2 Department of Neurosurgery, University Hospital Düsseldorf, 40225 Düsseldorf, Germany; igor.fischer@med.uni-duesseldorf.de (I.F.); rajiv.khajuria@med.uni-duesseldorf.de (R.K.)

**Keywords:** lifestyle-related risk factors, intracranial aneurysms, subarachnoid hemorrhage, prevention, rupture risk assessment

## Abstract

*Background and Objectives*: The rising incidence of modifiable lifestyle risk factors and cardiovascular diseases, driven by poor diet, inactivity, excessive alcohol use, and smoking, may influence the development and rupture of intracranial aneurysms (IA). This study aimed to examine the impact of lifestyle-related and cardiovascular risk factors on IA rupture and patient outcomes. *Materials and Methods*: We developed the “MARVIN” (Metabolic and Adverse Risk Factors and Vices Influencing Intracranial Aneurysms) model and conducted a retrospective analysis of 303 patients with 517 IAs, treated between 2007 and 2020. Of these, 225 patients were analyzed for rupture status and 221 for clinical outcomes. The analysis focused on hypertension, diabetes, hypercholesterolemia, vascular diseases, nicotine and alcohol abuse, obesity, aneurysm rupture status, and clinical outcomes. Logistic regression was used to evaluate the impact of these risk factors. *Results*: Among those with risk factors, 24.9% (56/225) and 25.3% (56/221) had one, 32.0% (72/225) and 30.8% (68/221) had two, 20.0% (45/225) and 20.4% (45/221) had three, 12.0% (27/225) and 12.2% (27/221) had four, 4.0% (9/225) and 4.1% (9/221) had five, 0.9% (2/225) had six in both groups, and 0.4% (1/225) and 0.5% (1/221) had seven risk factors, respectively. Strong relationships were found between lifestyle-related vascular risk factors, indicating multiple comorbidities in patients with unhealthy habits. Smokers with ruptured aneurysms had higher WFNS (World Federation of Neurosurgical Societies) scores, but nicotine abuse did not affect long-term outcomes. The most significant predictors for poor outcomes were WFNS score and age, while age and a history of vascular diseases were protective against rupture. Despite the high prevalence of modifiable risk factors, they did not significantly influence rupture risk. *Conclusions*: The findings suggest a need for multifactorial risk assessment strategies in managing IA patients. Future studies with larger cohorts are required to confirm these results and better understand IA progression.

## 1. Introduction

Intracranial aneurysms (IAs) are pathological dilations of the brain-supplying arteries. Although IAs can often remain asymptomatic for many years, they pose a significant risk of rupture. The estimated incidence of subarachnoid hemorrhage (SAH) is 6.7 per 100,000 persons per year, resulting in approximately 500,000 cases globally each year [1]. Despite advanced intensive care, SAH has a 30-day mortality rate of about 42%, with 10–25% of patients dying before reaching the hospital [2,3,4]. Additionally, up to 50% of survivors suffer from long-term impairments, requiring ongoing assistance in daily life [4,5,6,7].

The mechanisms leading to aneurysm rupture are not fully understood. Numerous extrinsic and intrinsic factors are associated with aneurysm rupture, including aneurysm size, location, configuration, surface characteristics, and hemodynamic properties [8,9]. Additionally, known cardiovascular risk factors such as arterial hypertension, nicotine abuse, and alcohol consumption contribute to aneurysm progression and eventual rupture [8]. These influencing factors are partially modifiable and include the following: heavy alcohol consumption, smoking, obesity, arterial hypertension, presence of diabetes mellitus, and hypercholesterolemia [10,11,12,13,14,15]. However, the specific impact of these risk factors remains unclear. While there is an agreement regarding an increased risk associated with alcohol consumption [10,13], smoking [11,12,13], and arterial hypertension [11,13,14,15], the data regarding the influence of overweight, diabetes, and hypercholesterolemia are inconsistent. Notably, some studies have identified a higher BMI as a protective factor, particularly in women [12,16]. Feigin demonstrated the protective effect of diabetes and hypercholesterolemia, which was corroborated by further studies [12,13,17].

On the other hand, the role of these risk factors in the development of cardiovascular diseases is well documented [18,19]. The Heart Disease and Stroke Statistics 2017 Update highlights key trends in cardiovascular health. Notably, mean LDL cholesterol levels among US adults have decreased, but the use of cholesterol-lowering treatments, including statins, has significantly increased, indicating a widespread incidence of this issue that is effectively managed with medication. Hypertension remains widespread, affecting 34% of US adults aged 20 and older. Additionally, around 23.4 million US adults have been diagnosed with diabetes, with another 7.6 million undiagnosed cases and 81.6 million people with prediabetes. Obesity rates among US adults and youth have risen markedly, and 15.2% of US adults used tobacco in 2015 [18]. Similar trends are observed in Germany, where the prevalence of overweight, obesity, and high blood pressure has increased. Smoking rates among women have risen by about 8%, particularly among younger women, while high cholesterol levels have shown a slight decrease [19]. This increase in modifiable lifestyle risk factors and cardiovascular diseases is largely attributed to the growing prevalence of unhealthy lifestyles, characterized by poor diet, physical inactivity, excessive alcohol consumption, and smoking. Consequently, these factors may also play a significant role in the pathophysiology of IA formation and rupture, as well as in the treatment of patients with IA.

In this study, we aimed to establish a stereotypical patient model, named “MARVIN” (Metabolic and Adverse Risk Factors and Vices influencing Intracranial aneurysms), which represents individuals with an unhealthy lifestyle and associated vascular diseases. This model underscores the typical risk factors and characteristics of patients whose lifestyles significantly contribute to vascular complications, emphasizing the importance of targeted lifestyle modifications in this population. This study furthermore aimed to investigate the extent to which modifiable lifestyle-related risk factors (such as smoking, heavy alcohol consumption, and obesity) and cardiovascular risk factors (including arterial hypertension, diabetes mellitus, hyperlipidemia, and vascular diseases), which are often associated with an unhealthy lifestyle, influence the rupture of IA and the clinical outcomes of patients with IA.

## 2. Materials and Methods

To conduct this study, we examined a previously collected database of patients at the Department of Neurosurgery of the Otto-von-Guericke University Hospital Magdeburg. The database included 303 patients who had been diagnosed with 517 saccular IAs and who had been presented to our department during the period from 2007 to 2020. IAs were diagnosed using computed tomography angiography, magnetic resonance angiography, or digital subtraction angiography. We conducted a retrospective analysis and applied the following inclusion criteria: the presence of complete data sets regarding the following lifestyle risk factors: arterial hypertension, diabetes type 2, hypercholesterolemia, vascular diseases of various organ systems, nicotine abuse, alcohol abuse, and obesity; the availability of information regarding the rupture status and the clinical outcome.

**Figure 1:**
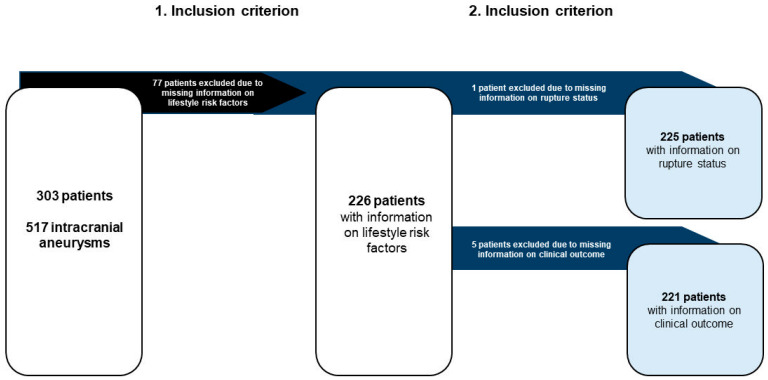
Presentation of the patient cohort according to the inclusion criteria of the study. Of 303 patients with IA, 226 had complete information on the lifestyle-associated risk factors recorded. Information on rupture status was available for 225 patients and on clinical outcomes for 221.

A total of 77 patients were excluded because of incomplete data on lifestyle-related risk factors. Vascular diseases were defined as peripheral arterial occlusive disease, stroke, myocardial infarction, and coronary artery stenosis. Furthermore, we excluded one patient due to missing information on rupture status and five patients due to the absence of clinical outcome data (Figure 1).

### 2.1. Data Acquisition

The clinical data for this study were obtained retrospectively through a comprehensive analysis of patient medical records, anamneses, medication registries, and existing diagnostic imaging. The evaluation of patient medical histories included the assessment of prevalent cardiovascular diseases, associated risk factors, and other critical health conditions, such as malignant neoplasms or significant autoimmune disorders requiring immunosuppressive therapy. Regarding the natural progression and management of IAs, extensive data were collected from the results of diagnostic imaging, aneurysm-specific risk factors, and assessments of patient clinical outcomes following aneurysm treatment or rupture. The relevant parameters are outlined and defined as follows:

The collected epidemiological data included the age of the patient at the time of diagnosis and their biological sex. Pre-existing conditions and risk factors were also assessed, including hypertension, defined by a documented diagnosis or the use of antihypertensive medications [14], and diabetes mellitus, diagnosed as type 2 diabetes or the use of oral antidiabetic drugs or insulin. Hyperlipidemia was documented either by diagnosis or through the use of medication to lower blood lipid or cholesterol levels. Other vascular diseases, such as peripheral arterial disease, stroke, myocardial infarction, and coronary artery stenosis, were included in the analysis. Obesity was defined as a body mass index greater than 30 kg/m^2^, while nicotine use encompassed both current and former users [13,14]. Alcohol use was classified as the consumption of more than 50 g of alcohol per week [13].

Aneurysm-specific factors were considered, including aneurysm rupture, which was determined based on intra-operative findings, imaging results, or CT evidence of hemorrhage patterns. Aneurysm multiplicity was defined as the presence of two or more intracranial aneurysms [20], and aneurysm localization was assessed through angiographic evaluation. Clinical scores used in the study included the WFNS (World Federation of Neurosurgical Societies) score, which classifies SAH based on the Glasgow Coma Scale and the presence of focal neurological deficits [21]. The Fisher Grade was employed to classify the severity of SAH based on CT scan findings, ranging from no visible blood to large intracerebral or intraventricular clots [22]. Finally, the Modified Rankin Scale at discharge was used to assess neurological and functional impairment, with scores ranging from no symptoms to death [23].

### 2.2. Statistical Analysis

In this study, we considered patient age, sex, initial WFNS score, and various lifestyle-related factors—hypertension, nicotine abuse, diabetes type 2, hypercholesterolemia, obesity, alcohol abuse, and a history of vascular diseases—as independent variables. The dependent variables were the patient outcomes, measured by the mRS score, and the occurrence of IA rupture. The mRS was categorized into three groups: favorable outcome (mRS = 0–2), poor outcome (mRS = 3–5), and death (mRS = 6).

To ensure data plausibility and avoid collinearities, we examined the relationships between the dependent variables both statistically and visually. Dichotomous variables were tested for independence using the χ^2^ test, while the relationship between dichotomous and continuous variables was assessed using the *t*-test.

We modeled the influence of independent variables on rupture using logistic regression, and on patient outcomes using proportional odds logistic regression (POLR). Initially, simple regressions were performed for each dependent variable. The significance level used for all tests was set at the standard threshold of 0.05. Subsequently, variables that were found to be significant were included in multiple regression analyses.

## 3. Results

### 3.1. Cohort Overview

#### 3.1.1. Rupture Analysis

The cohort for the rupture analysis consisted of 225 patients, with a mean age of 54.8 years. Among these patients, 33.3% were male (n = 75) and 66.7% were female (n = 150). Arterial hypertension was present in 73.3% of the patients, highlighting its significant prevalence. Nicotine abuse was also notably high, affecting 62.7% of the patients. Obesity was identified in 29.8% of the cohort, while hypercholesterolemia was noted in 20.0%. Diabetes type 2 affected 12.9% of the patients, and vascular diseases were reported in 11.6%. Alcohol abuse was observed in 16.0% of the patients (Table 1).

**Figure 2:**
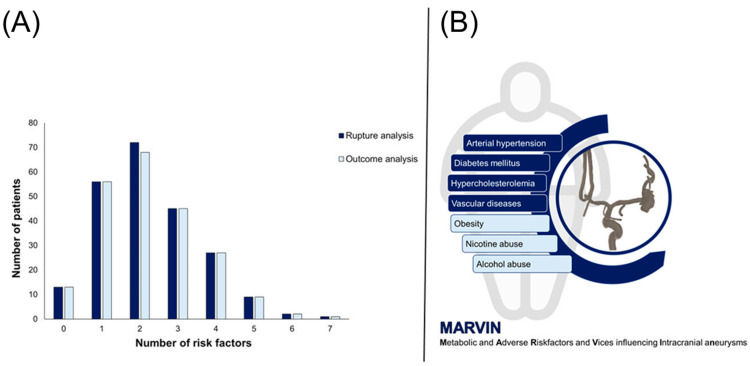
Part (**A**) of the figure shows the distribution of the number of risk factors for the rupture and outcome analyses, with the rupture analysis shown in dark blue and the outcome analysis in light blue. Part (**B**) of the figure schematically shows the concept of “MARVIN”, which stands for “Metabolic and Adverse Risk Factors and Vices influencing Intracranial aneurysms” and shows the accumulation of the established seven risk factors in patients who lead an unhealthy lifestyle.

The distribution of the number of lifestyle-related risk factors among the patients was as follows. A small group of patients, 5.8%, had no risk factors. One risk factor was present in 24.9% of the patients, while 32.0% had two risk factors. A total of 20.0% of the patients had three risk factors, and 12.0% had four risk factors. Only 4.0% of the patients had five risk factors, and an even smaller percentage had six (0.9%) and seven (0.4%) risk factors (Figure 2).

A total of 154 patients presented with SAH, and 88 patients had multiple aneurysms. The majority of IAs were found in the anterior communicating artery (ACOM), affecting 46.7% of the patients. The middle cerebral artery (MCA) was the next most common site, with 20.0% of the patients having aneurysms in this location. Aneurysms in the internal carotid artery (ICA) were identified in 12.4% of the patients, while 11.1% had aneurysms in the basilar artery. Other locations included the posterior inferior cerebellar artery (PICA), with 3.1% of the patients, and the posterior communicating artery (PCOM), vertebral artery, and superior cerebellar artery (SCA), each affecting 1.3% of the patients. Less frequently, aneurysms were located at the anterior cerebral artery (ACA), pericallosal artery, and anterior choroidal artery, each affecting 0.9% of the patients. The mean diameter of the IA in this cohort was 7.4 mm (Table 1).

The distribution of the WFNS scores among the analyzed ruptured cases was as follows: WFNS 1 in 86 cases (55.8%), WFNS 2 in 23 cases (14.9%), WFNS 3 in 4 cases (2.6%), WFNS 4 in 21 cases (13.6%), and WFNS 5 in 18 cases (11.7%). Regarding the Fisher grades, 4 cases (2.6%) were classified as grade 1, 5 cases (3.3%) as grade 2, 55 cases (35.7%) as grade 3, and 89 cases (55.8%) as grade 4 (Table 1).

#### 3.1.2. Outcome Analysis

The cohort for the outcome analysis consisted of 221 patients, with a mean age of 54.9 years, including 145 females (65.6%) and 76 males (34.4%). Arterial hypertension was present in 73.8% of the patients, while 13.1% had diabetes type 2, and 20.4% had hypercholesterolemia. A history of vascular diseases was noted in 11.8% of the patients. Nicotine abuse was reported in 62.4%, alcohol abuse in 16.3%, and obesity in 29.0% of the cohort (Table 1).

The distribution of the number of lifestyle-related risk factors per patient varied. Specifically, 5.9% of the patients had no risk factors, 25.3% had one, and 30.8% had two. Additionally, 20.3% of the patients had three risk factors, 12.2% had four, 4.1% had five, 0.9% had six, and 0.5% had seven risk factors (Figure 2).

Among the 221 patients, 152 presented with SAH, and 87 had multiple IAs. The majority of IAs were found at the ACOM, comprising 46.6% of the cases. The MCA was the next most common site, accounting for 20.4% of the cases. IAs in the ICA were identified in 12.2% of patients, while 10.9% had IAs in the basilar artery. IAs were less frequently located in the posterior inferior cerebellar artery, the PCOM, the vertebral artery, the ACA, the pericallosal artery, the anterior choroidal artery, and the superior cerebellar artery (Table 1). The mean diameter of the IA in this cohort was 7.4 mm (Table 1).

For the outcome analysis, in ruptured cases, the distribution of the WFNS score and the Fisher grade was as follows: WFNS scores included 86 cases (56.6%) with WFNS 1, 23 cases (15.1%) with WFNS 2, 4 cases (2.6%) with WFNS 3, 21 cases (14.8%) with WFNS 4, and 18 cases (11.8%) with WFNS 5. Regarding Fisher grades, the distribution was 4 cases (2.6%) with grade 1, 5 cases (3.3%) with grade 2, 54 cases (35.5%) with grade 3, and 89 cases (56.6%) with grade 4 (Table 1).

### 3.2. Analysis of Clinical Outcome and Rupture

**Figure 3:**
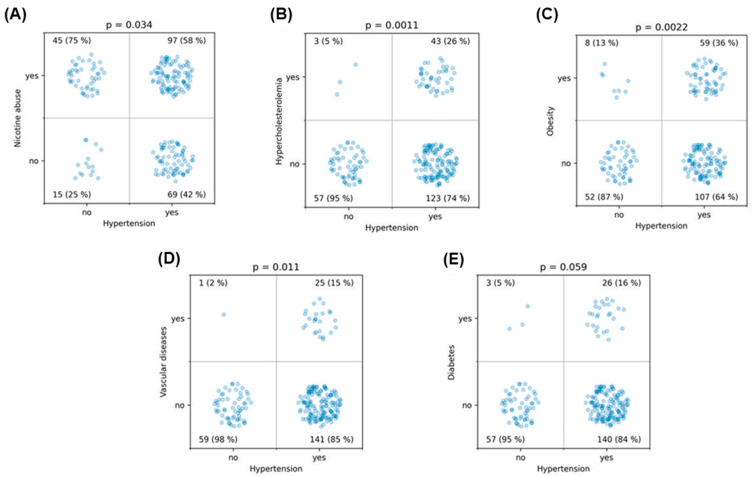
This figure illustrates the associations between various lifestyle-associated risk factors and hypertension, tested using the χ^2^ test: (**A**) shows the association between hypertension and nicotine abuse, (**B**) between hypercholesterolemia and hypertension, (**C**) between obesity and hypertension, (**D**) between a history of vascular diseases and hypertension, and (**E**) between diabetes and hypertension.

**Figure 4:**
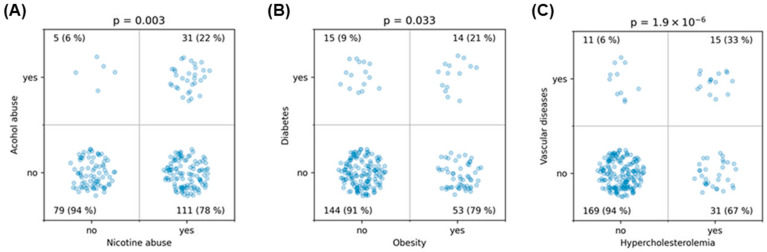
This figure illustrates the associations tested using the χ^2^ test between (**A**) alcohol and nicotine abuse, (**B**) diabetes and obesity, and (**C**) history of vascular diseases and hypercholesterolemia.

**Figure 5:**
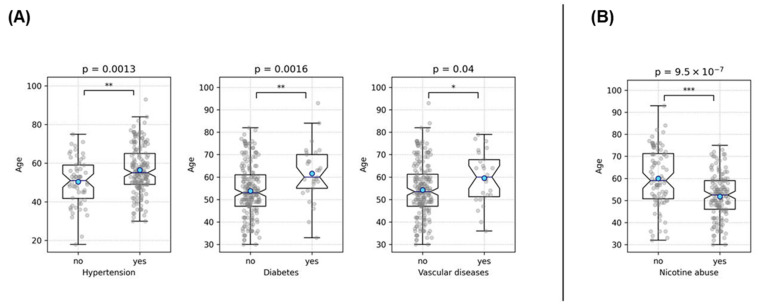
This figure illustrates the associations between age and various risk factors examined using a *t*-test. Part (**A**) depicts the association between older age and the increased likelihood of hypertension, diabetes, and a history of vascular diseases. Part (**B**) shows the association between younger age and a higher prevalence of nicotine abuse. Asterisks denote the level of statistical significance: * = *p* < 0.05, ** = *p* < 0.01, and *** = *p* < 0.001. Blue dots represent the mean values.

Initially, the independent variables were examined in detail for potential associations. Hypertension was associated with nicotine abuse (*p* = 0.034), hypercholesterolemia (*p* = 0.0011), obesity (*p* = 0.0022), history of vascular diseases (*p* = 0.011), and showed a trend with diabetes (*p* = 0.059) (Figure 3, Table 2). There was a strong association between nicotine and alcohol abuse (*p* = 0.003). Obesity was associated with diabetes (*p* = 0.033), and hypercholesterolemia was highly significantly associated with a history of vascular diseases (*p* < 10^−5^) (Figure 4, Table 2). These findings demonstrate strong interrelationships among common lifestyle-associated vascular risk factors, supporting our hypothesis of a stereotypical unhealthy patient with multiple resultant vascular diseases and emphasizing the validity of our data. Furthermore, older patients were more likely to have hypertension (*p* = 0.0013), diabetes (*p* = 0.0016), and a history of vascular diseases (*p* = 0.04) (Figure 5). Interestingly, younger patients were more likely to be associated with nicotine abuse (*p* < 10^−6^) (Figure 5). The gender of the patients had no influence in any of the analyses

While some independent variables were correlated, most of these variables did not have a significant impact on the primary outcomes (clinical outcome and rupture) and were thus excluded from the multiple regression model.

**Figure 6:**
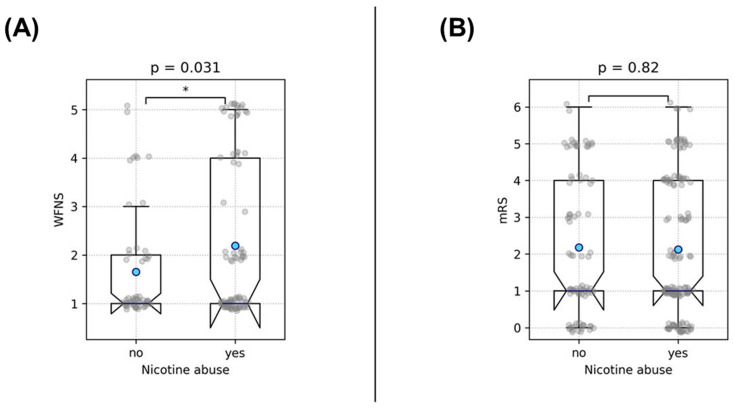
This figure illustrates the association between nicotine abuse and clinical outcomes in patients with ruptured IA, examined by a *t*-test. Part (**A**) shows that smokers presented with significantly higher WFNS scores at admission, indicating more severe initial clinical conditions. However, Part (**B**) demonstrates that nicotine abuse did not associate with a worse outcome at discharge, despite the more severe initial presentation. Asterisks denote the level of statistical significance: * = *p* < 0.05. Blue dots represent the mean values.

The analysis of the impact of the examined risk factors on the initial clinical presentation after aneurysm rupture revealed that smokers presenting with ruptured aneurysms were initially more severely affected, exhibiting WFNS scores approximately half a grade higher (*p* = 0.031). Interestingly, nicotine abuse did not correlate with the eventual patient outcome (trichotomized mRS, *p* = 0.8), indicating that despite the initially higher WFNS scores, the outcomes in our cohort were not worse compared to patients who were not smoking (Figure 6).

**Figure 7:**
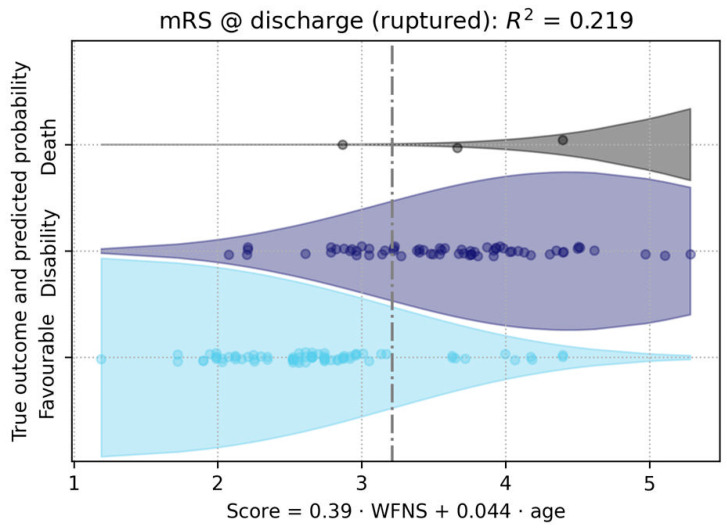
This figure illustrated the prediction of clinical outcome trichotomized in favorable outcome, disability, or death (proportional odds logistic regression). Here, the only significant predictors for a worse outcome were the WFNS score and age. The two are combined into a composite score, as given by the regression model. The x-axis represents the composite score, which is calculated using the formula provided below the axis.

In the analysis of clinical outcomes, measured by the mRS, age (OR = 1.02, 95% CI [1.007–1.034], *p* = 0.003) and WFNS score (OR = 1.4, 95% CI [1.2–1.6], *p* < 0.001) emerged as significant predictors in the univariate regression. Both variables were included in the subsequent multivariate regression model. In the final model, the WFNS score (*p* < 10⁻⁶) and age (*p* < 10⁻⁵) both remained significant predictors of a worse clinical outcome (Figure 7, Table 3).

Other variables, including sex (*p* = 0.6), hypertension (*p* = 0.6), nicotine abuse (*p* = 0.8), diabetes (*p* = 0.1), hypercholesterolemia (*p* = 0.4), obesity (*p* = 0.1), alcohol abuse (*p* = 0.7), and vascular diseases (*p* = 0.5), did not show significant associations with clinical outcomes (Table 3).

**Figure 8:**
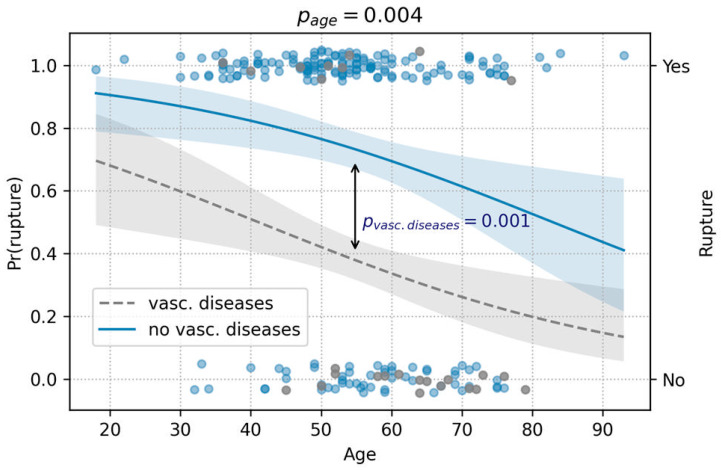
The prediction analysis of rupture, depicted in this figure, showed protective effects of higher age and history of vascular diseases against rupture (logistic regression). Pr(rupture) represents the conditional probability of rupture, based on the patient’s age and history of vascular diseases.

In the analysis of factors associated with aneurysm rupture, age (OR = 0.96, 95% CI [0.94–0.98], *p* = 0.001), and a history of vascular diseases (OR = 0.2, 95% CI [0.08–0.47], *p* = 0.0002) emerged as the only significant predictors in the univariate regression. Consequently, both variables were included in the subsequent multivariate regression model. The results demonstrated that age was inversely associated with the risk of rupture (*p* = 0.004), while a history of vascular diseases significantly reduced the odds of rupture (*p* = 0.001) (Figure 8, Table 4).

Other variables assessed in the model, including sex (*p* = 0.3), the WFNS score (*p* = 1), hypertension (*p* = 0.5), nicotine abuse (*p* = 0.3), diabetes (*p* = 0.7), hypercholesterolemia (*p* = 0.09), obesity (*p* = 1), and alcohol abuse (*p* = 0.9), were not found to be significant (Table 4).

## 4. Discussion

In this study, we aimed to investigate the extent to which modifiable lifestyle-related risk factors (such as smoking, heavy alcohol consumption, and obesity) and cardiovascular risk factors (including arterial hypertension, diabetes mellitus, hyperlipidemia, and vascular diseases) influence the rupture of IAs and clinical outcomes of affected patients. To visualize our hypothesis, we establish a stereotypical patient model named “MARVIN” to represent individuals with an unhealthy lifestyle and associated vascular diseases. This model highlights the typical risk factors and characteristics of patients whose lifestyles significantly contribute to vascular complications, emphasizing the importance of targeted lifestyle modifications.

Our analysis revealed that vascular risk factors were highly prevalent in the patient cohort. Arterial hypertension was present in 73.3% of the patients, highlighting its significant prevalence. Among the modifiable risk factors, nicotine abuse stood out, affecting 62.7% of the patients, making it a significant target for prevention efforts. Additionally, obesity was present in 29.8% of the patients, hypercholesterolemia in 20.0%, type 2 diabetes in 12.9%, and vascular diseases, including coronary issues, in 11.6%. Alcohol abuse was identified in 16.0% of the cohort. Upon examining the independent variables, we found strong associations between several risk factors. Hypertension was commonly linked with nicotine abuse, hypercholesterolemia, obesity, and a history of vascular diseases. Nicotine and alcohol abuse showed a strong interrelationship. Obesity was frequently associated with diabetes, and hypercholesterolemia was significantly linked to a history of vascular diseases. These findings demonstrate the robust interrelationships among common lifestyle-associated vascular risk factors, further supporting our hypothesis of a stereotypical unhealthy patient with multiple resultant vascular diseases.

The significance of these risk factors is also evident in the numerous scores used to assess the risk of IA rupture. For example, the PHASES score awards an additional point to patients with arterial hypertension [14]. The New Treatment score, published by Juvela, specifically highlights ongoing nicotine abuse as a critical risk factor [24,25]. In the UIATS, which balances the risk of treatment against the risk of spontaneous progression based on various criteria, smoking, alcohol abuse, and arterial hypertension are key considerations. According to UIATS, patients with these risk factors are more likely to be recommended for treatment [6]. To emphasize the importance of these factors, smoking cessation and control of arterial hypertension are clearly advised upon the diagnosis of an incidental aneurysm [8]. Conversely, the influence of risk factors such as overweight, diabetes, and hypercholesterolemia remains unclear, with some authors suggesting protective effects [12,13,16,17].

Besides lifestyle-related risk factors, patient age is also a significant risk factor, with numerous studies demonstrating a link between older age and increased rupture rates [12,14]. In our study, IA rupture was inversely associated with older age and the presence of vascular diseases, indicating that older patients and those with vascular diseases were less frequently affected by rupture. This finding contrasts with the results described in the literature. Due to the retrospective nature of this study, one possible explanation for the apparent protection conferred by cardiovascular diseases and older age is that these patients may undergo cranial imaging more frequently due to nonspecific symptoms or their known conditions. This leads to the detection and subsequent treatment of intracranial aneurysms before rupture, thus classifying them as “unruptured” in our analysis. The natural course without such interventions remains unclear in these cases. Interestingly, our study found no significant impact of lifestyle-associated risk factors on the likelihood of rupture. A noteworthy aspect of our findings is that smokers who experienced a rupture were initially more severely affected clinically. However, this was not reflected in the clinical outcomes, indicating that despite higher initial WFNS scores, the final outcomes in our cohort were not worse compared to non-smoking patients. In our study, the clinical outcome was influenced only by the WFNS score and the patient’s age, with younger patients exhibiting better clinical outcomes.

Our findings further reveal that most patients (156 vs. 67) had more than one vascular risk factor, with most exhibiting two risk factors. This supports our “MARVIN” hypothesis, indicating that many patients harbor multiple risk factors and should be considered “vascularly unhealthy.” Conversely, there are “otherwise vascularly healthy” aneurysm patients who might require separate consideration.

Research on coronary heart disease (CHD) has already clarified the quantitative relationship between these lifestyle-related risk factors and CHD risk through the Framingham Heart Study and other investigations [26,27]. These studies have shown that the major risk factors for CHD are additive in their predictive power, meaning that the total risk for an individual can be calculated by summing the contributions of each major risk factor.

In response to the high prevalence of these risk factors, the American Heart Association (AHA) has identified seven key health metrics, or “ideal health factors,” to help reduce the future burden of cardiovascular disease [28]. These metrics encompass regular physical activity, maintaining a normal BMI, adhering to a healthy diet, achieving low serum cholesterol levels, sustaining normal blood pressure, maintaining low fasting plasma glucose levels, and abstaining from smoking. Achieving these ideal health factors is associated with lower levels of biomarkers for subclinical atherosclerosis, such as carotid intima-media thickness [29,30], and is linearly linked to the risk of developing cardiovascular disease, as well as both all-cause and cardiovascular mortality [31,32,33]. Attaining ideal levels of these key cardiovascular health factors is crucial for reducing the risk of premature cardiovascular-related death [31]. Unfortunately, very few people, including children, achieve an ideal health profile [18].

Given this background, a logical next step in understanding the natural course of patients with IA is to closely examine vascular risk factors and their specific impacts on risk. This approach could provide deeper insights into the progression and management of IA, potentially mirroring the comprehensive risk assessment models established for CHD. Considering the high prevalence of multiple vascular risk factors among IA patients, it is crucial to adopt a multifactorial risk assessment strategy. This strategy could help identify patients at higher risk and tailor individualized treatment plans, ultimately improving patient outcomes.

This study has several limitations that must be acknowledged. Firstly, the retrospective nature of our analysis introduces inherent biases, as it relies on previously collected data, which may not comprehensively capture the natural course of treated incidental aneurysms. The lack of prospective follow-up data limits our ability to observe the progression of these aneurysms over time, which is crucial for understanding the long-term impact of the identified risk factors. Secondly, due to the observational design of the study, we cannot draw direct causal relationships from our results. While associations between certain risk factors and aneurysm rupture were identified, causality cannot be established without more rigorous, controlled studies. Additionally, the relatively small cohort size, particularly the limited number of patients with multiple risk factors, reduces the statistical power and generalizability of our findings. While our study analyzed 303 patients, future studies with larger sample sizes are necessary to draw more robust conclusions, particularly concerning the interplay of multiple risk factors. Another limitation is the absence of significant associations between modifiable lifestyle-related factors, such as hypertension, nicotine abuse, and obesity, and the risk of aneurysm rupture. Despite their high prevalence, these factors did not significantly affect rupture risk in our cohort. This suggests that other, potentially unrecognized factors may play a more substantial role, and further investigation is needed to identify these determinants. Moreover, our study did not include long-term follow-up data after patient discharge, which limits our ability to assess the true impact of modifiable risk factors and treatments on patient outcomes over time. Future research incorporating extended follow-up periods is essential to understand the long-term implications of these factors and the potential benefits of early interventions. While we analyzed conditions such as hypertension and diabetes, we did not explore how medications, including antihypertensives or statins, might have influenced outcomes. This omission represents an important gap in our study, as these treatments could potentially affect the risk of aneurysm rupture or modify clinical outcomes. Future studies should address the role of these medications to provide a more comprehensive understanding of the relationships between medical management and aneurysm rupture. Finally, while the MARVIN model was conceptually useful in our analysis, it has not been validated with experimental or clinical data to confirm its predictive value for aneurysm rupture risk. Further validation through experimental studies or clinical trials is required to confirm its utility and to establish its potential application in clinical practice.

## 5. Conclusions

This study highlights the significant prevalence of modifiable lifestyle-related and cardiovascular risk factors among patients with IA. Arterial hypertension and nicotine abuse were particularly prevalent and most patients had more than one risk factor, supporting the “MARVIN” model of a stereotypical patient with multiple risk factors. Despite strong associations with IA presence, these factors did not affect the likelihood of rupture. Interestingly, the inverse relationship between rupture and both older age and existing vascular diseases suggests that these groups may undergo more frequent incidental detection and treatment, classifying their aneurysms as “unruptured” in our analysis. The analysis of clinical outcomes in our cohort revealed that smokers presenting with ruptured IA were initially more severely affected, with higher WFNS scores, but their final outcomes were not worse compared to non-smokers. The only significant predictors of worse outcomes were the WFNS score and patient age. These findings emphasize the need for multifactorial risk assessment strategies to better identify and manage high-risk patients. Future prospective studies with larger cohorts are essential to validate these results and further explore the natural progression of untreated incidental IA.

## Figures and Tables

**Table 1:** Presentation of the demographic data for the analysis of the rupture and the clinical outcome.

Parameter	Rupture Analysis (n = 225)	Outcome Analysis (n = 221)
Sex	75 male; 150 female	76 male; 145 female
Age at diagnosis (mean)	54.8 (±12.5) years	54.9 (±12.5) years
Arterial hypertension	165 (73.3%)	163 (73.8%)
Diabetes mellitus Type 2	29 (13%)	29 (13.1%)
Hypercholesterolemia	45 (20%)	45 (20.4%)
Vascular diseases	26 (11.6%)	26 (11.8%)
Nicotine abuse	141 (62.7%)	138 (62.4%)
Alcohol abuse	36 (16%)	36 (16.3%)
Obesity	67 (30%)	64 (29.0%)
Ruptured aneurysms	154 (68.4%)	152 (68.8%)
Multiple aneurysms	88 (39.1%)	87 (39.4%)
Aneurysm size (mm, mean)	7.4 (±3.0)	7.4 (±3.0)
Aneurysm localization		
ACA	2 (0.9%)	2 (0.8%)
ACOM	105 (46.7%)	103 (46.6%)
Pericallosal artery	2 (0.9%)	2 (0.8%)
MCA	45 (20%)	45 (20.4%)
ICA	28 (12.4%)	27 (12.2%)
PCOM	3 (1.3%)	3 (1.4%)
Anterior choroidal artery	2 (0.9%)	2 (0.8%)
Basilar artery	25 (11.1%)	24 (10.9%)
Vertebral artery	3 (1.3%)	3 (1.4%)
PICA	7 (3.1%)	7 (3.2%)
SCA	3 (1.3%)	3 (1.4%)
WFNS score		
1	86 (55.8%)	86 (56.6%)
2	23 (14.9%)	23 (15.1%)
3	4 (2.6%)	4 (2.6%)
4	21 (13.6%)	21 (14.8%)
5	18 (11.7%)	18 (11.8%)
No information	2 (1.3%)	0 (0%)
Fisher grade		
1	4 (2.6%)	4 (2.6%)
2	5 (3.3%)	5 (3.3%)
3	55 (35.7%)	54 (35.5%)
4	89 (55.8%)	89 (56.6%)
No information	1 (0.7%)	0 (0%)

ACA: anterior cerebral artery; ACOM: anterior communicating artery; MCA: middle cerebral artery; ICA: internal carotid artery; PCOM: posterior communicating artery; PICA: posterior inferior cerebellar artery; SCA: superior cerebellar artery.

**Table 2:** Associations between hypertension and other risk factors, including *p*-values and odds ratios, highlighting the significant correlations identified in the detailed analysis of independent variables.

Variable 1	Variable 2	*p*-Value	Odds Ratio
Hypertension	Nicotine abuse	0.034	0.5
Hypertension	Hypercholesterolemia	0.001	6.6
Hypertension	Obesity	0.002	3.6
Hypertension	Vascular diseases	0.011	10.5
Hypertension	Diabetes	0.059	3.5
Nicotine abuse	Alcohol abuse	0.003	4.4
Hypercholesterolemia	Vascular diseases	1.86 × 10^−6^	7.4
Obesity	Diabetes	0.033	2.5

**Table 3:** Univariate and multivariate analysis of variables associated with clinical outcomes. Bold *p*-values indicate statistically significant results (*p* < 0.05). Significant predictors in both univariate and multivariate analyses include age and WFNS score, while other variables did not reach statistical significance in either model.

Variable	Univariate Analysis (*p*-Value)	Multivariate Analysis (*p*-Value)
Age	**0.003**	**<10⁻⁵**
Sex	0.6	-
WFNS Score	**<0.001**	**<10⁻⁶**
Hypertension	0.6	-
Nicotine abuse	0.8	-
Diabetes	0.1	-
Hypercholesterolemia	0.4	-
Obesity	0.1	-
Alcohol abuse	0.7	-
Vascular diseases	0.5	-

**Table 4:** Univariate and multivariate analysis of variables associated with aneurysm rupture. Bold *p*-values indicate statistically significant results (*p* < 0.05). Age and a history of vascular diseases were significant predictors in both univariate and multivariate analyses, while other variables did not show significant associations with aneurysm rupture.

Variable	Univariate Analysis (*p*-Value)	Multivariate Analysis (*p*-Value)
Age	**0.001**	**0.004**
Sex	0.3	-
Hypertension	0.5	-
Nicotine abuse	0.3	-
Diabetes	0.7	-
Hypercholesterolemia	0.09	-
Obesity	1	-
Alcohol abuse	0.9	-
Vascular diseases	**0.0002**	**0.001**

## Data Availability

The data supporting the findings of this study are not openly available due to reasons of sensitivity and are available from the corresponding author upon reasonable request.
